# Complications and Salvage Management in Ilizarov Reconstruction After Wide Excision for Distal Femoral Giant Cell Tumour: A Retrospective Series of 10 Cases

**DOI:** 10.7759/cureus.107972

**Published:** 2026-04-29

**Authors:** Jishnu Prakash Baruah, Shyam Sunder

**Affiliations:** 1 Orthopaedics, Assam Medical College and Hospital, Dibrugarh, IND; 2 Orthopaedics, Pondicherry Institute of Medical Sciences, Puducherry, IND

**Keywords:** distal femur tumors, distraction osteogenesis (do), giant cell tumour of bone, ilizarov fixator, limb reconstruction

## Abstract

Background

Wide excision for Campanacci Grade III giant cell tumours (GCT) of the distal femur results in major segmental defects. Where endoprosthetic reconstruction is limited, Ilizarov distraction osteogenesis provides a biologic limb salvage option, though with frequent mechanical and infectious complications.

Objectives

The objective of this study was to describe clinical outcomes, complication profiles, management strategies, and salvage interventions in 10 consecutive cases of wide resection followed by Ilizarov reconstruction for distal femoral GCT in young adults.

Materials and methods

This retrospective review included 10 consecutive eligible patients aged 20-38 years with histologically confirmed Campanacci III distal femur GCT, treated between 2013 and 2020 at a single tertiary referral centre. Inclusion required a segmental defect of at least 8 cm after resection and a minimum of 24 months’ follow-up; patients with pre-study recurrence or systemic bone disorders were excluded (2/12 screened). Data collected included defect size, regenerate formation, union time, complications, interventions, and Musculoskeletal Tumour Society (MSTS) scores, using the standard 30-point system.

Results

The mean defect length was 13.5 cm (range 12-16 cm). Regenerate formation averaged 10.9 cm, with docking-site union achieved in all patients at a mean of 6.2 months (range 5-8 months). Major complications included varus deformity (4/10), pin-tract infection (3/10), docking-site non-union (2/10), fluid collection (3/10), and limb length discrepancy >3 cm (2/10). One non-union required bone grafting and Rail Fixator revision because of patient non-compliance; this was considered a failure of the primary Ilizarov construct but a successful salvage outcome. All patients ultimately achieved union and independent ambulation; the mean MSTS score was 23/30 (range 21-26) at a mean follow-up of 34 months.

Conclusion

Ilizarov reconstruction after distal femoral GCT excision can achieve limb salvage in selected young adults when alternatives are unavailable. Complications are common but usually manageable with close follow-up, timely intervention, and multidisciplinary care. Larger studies are needed to confirm these findings.

## Introduction

Giant cell tumour (GCT) of bone is a locally aggressive primary neoplasm that accounts for a notable fraction of benign bone tumours in young adults, with the distal femur being among the most frequently affected sites [1,2]. Treatment for Campanacci Grade III lesions necessitates wide en bloc resection to reduce recurrence risk, typically resulting in large metaphyseal bone defects. In resource-constrained environments, financial and technical challenges often preclude the use of endoprosthetic and allograft-based reconstruction [3]. In such settings, biologic reconstruction by distraction osteogenesis using circular external fixation, commonly known as the Ilizarov method, has been increasingly used [4].

While robust in principle, Ilizarov bone transport, especially in the femur, is accompanied by significant mechanical, infectious, and psychosocial challenges [5]. Given the unique anatomical, biomechanical, and functional demands of the femur, complications are common and may impact union rates, ambulatory capacity, and quality of life. Understanding these issues is imperative for clinicians aiming to optimize patient selection, surgical technique, and long-term outcomes.

This retrospective study describes clinical outcomes, complication profiles, management strategies, and salvage interventions in ten consecutive eligible patients with Campanacci III distal femoral GCTs treated by wide resection and Ilizarov reconstruction at a single tertiary center (2013-2020). We report transparent union rates, failure definitions, and functional scores using standard metrics to inform surgical decision-making in similar resource-constrained contexts.

## Materials and methods

Study design

This retrospective cohort study included 10 consecutive eligible patients with histologically confirmed Campanacci Grade III GCT of the distal femur, treated with wide en-bloc resection and Ilizarov reconstruction at the Department of Orthopaedics, Assam Medical College Hospital between January 2013 and December 2020. Institutional Ethics Committee (H) of the Assam Medical College approved the study (approval number: IEC/No 2026/AMC/EC/259) and, due to the nature of the study, waived the need for informed consent for use of retrospective de-identified data analysis.

Study population

Inclusion criteria were: (i) wide resection yielding ≥8 cm segmental defect, (ii) primary Ilizarov ring fixator with bone transport, and (iii) minimum 24-month follow-up from frame removal. Of 12 patients screened post biopsy confirmation, two were excluded before enrollment (one prior recurrence, one systemic bone disorder), yielding 10 consecutive eligible cases with 0% attrition. No patients were lost to follow-up after inclusion, and all consecutive eligible cases were analysed for primary reconstruction outcomes.

Surgical technique

**Figure 1:**
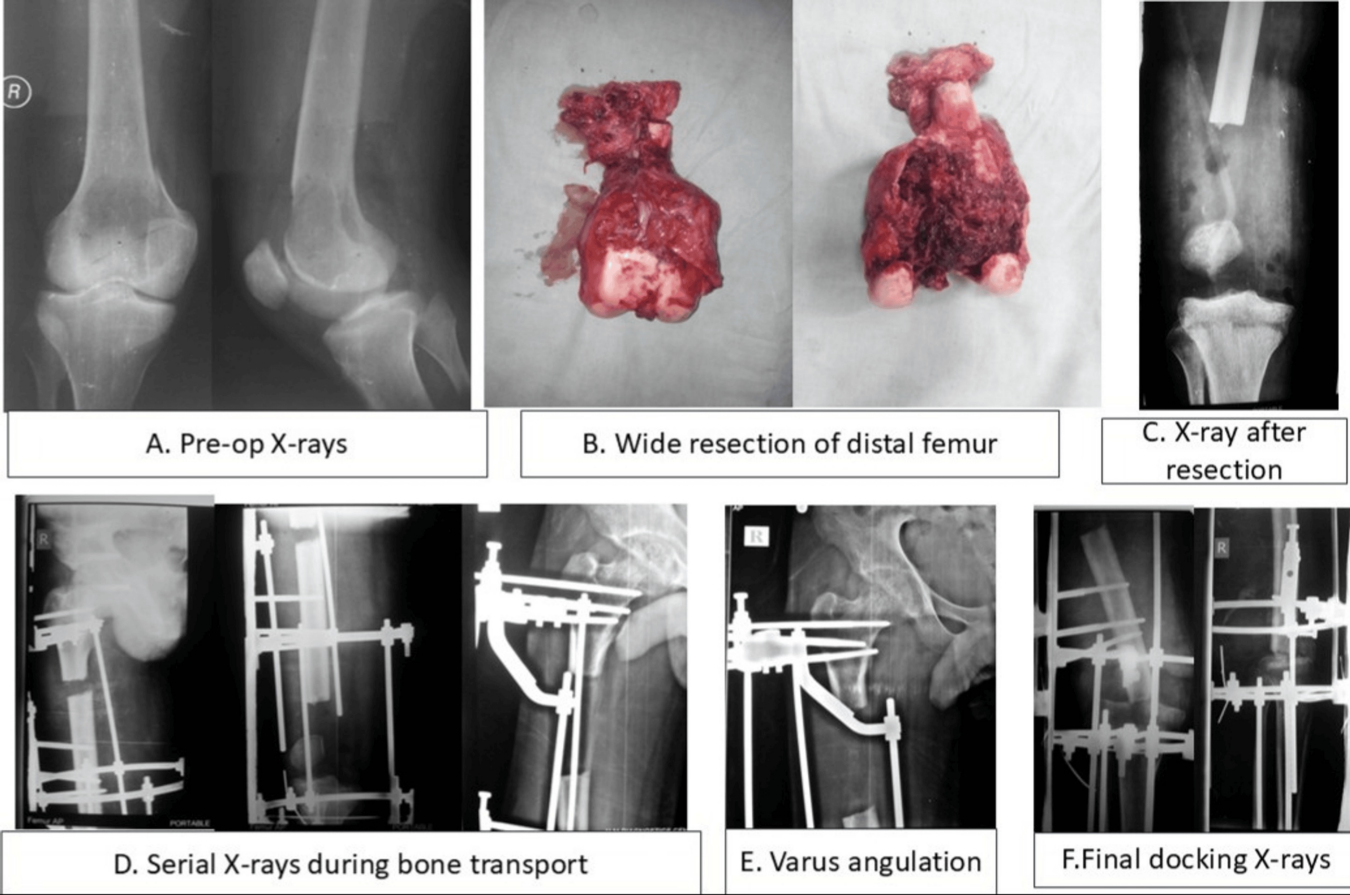
Grade III GCT distal femur managed with wide resection and Ilizarov fixator followed by bone transport A. Preoperative X-rays; B. Intra-op resection specimen; C.X-ray after resection; D. Serial X-rays during bone transport; E. Varus angulation of the femur; F. Final docking X-rays

All procedures were performed or directly supervised by the senior author (JPB). Wide en-bloc excision was achieved with clear margins based on preoperative MRI and intraoperative fluoroscopic guidance (Figures 1A, 1B). The resection cavity was copiously irrigated with hydrogen peroxide, followed by normal saline. Posterior neurovascular structures were protected with surgical mops during resection [5]. Post-resection defect length was measured intraoperatively with a calibrated ruler. Three patients received pre-assembled Ilizarov ring fixators at the index surgery (initial 4 cm docking achieved); seven patients were managed with rigid knee spanning braces to allow partial spontaneous soft tissue-mediated gap closure (2-3 mm) before delayed frame application (mean 10 days post-resection) (Figure 1C) [5].

Frames consisted of three to four rings proximally and distally, stabilized with 1.8 mm tensioned olive wires and 6 mm half-pins. A proximal metaphyseal-diaphyseal low-energy corticotomy was performed using multiple drill holes and an osteotome. After a latent period of 7-10 days, distraction was performed at 1 mm/day in four 0.25 mm increments (Figure 1D). Docking achieved via controlled transport with serial biplanar radiographic correction. At docking, axial compression was applied at 0.25 mm/day (Figures 1E, 1F). Autogenous iliac crest bone grafts were used prophylactically at the docking site for sclerotic metaphyseal ends in selected cases (n=2).

Weight-bearing progression was initiated from toe-touch during active transport to partial weight-bearing (20-40 kg) with crutches once three cortical regenerate columns were visible radiographically and full weight-bearing after docking site union confirmation. (Figure 1).

Postoperative care and rehabilitation

Postoperative care included daily chlorhexidine pin-site cleaning, fortnightly clinical assessment, intravenous cefuroxime for 48 hours followed by oral cefuroxime axetil for five days, and structured physiotherapy beginning on postoperative day 3. Monthly psychological counselling was provided to support compliance and coping.

Data collection

Data extracted from electronic medical records, operative notes, and serial radiographs: age, sex, defect length, regenerate length, external fixation index (months/cm regenerate), time to docking union, complications, reoperations, and frame removal date.

Complication definitions and measurement methods

Varus deformity was defined as >5° mechanical axis deviation measured monthly by the senior surgeon on anteroposterior hip-knee-ankle radiographs using Picture Archiving and Communication System (PACS) software. Docking-site non-union was defined as persistent radiolucency >3 mm at six months post-docking. Limb length discrepancy was defined as >3 cm shortening on scanograms. Pin-tract infection was classified clinically as soft tissue infection or osteomyelitis. Docking-site fluid collections were diagnosed as >10 mm fluid layers on postoperative ultrasound. The Musculoskeletal Tumour Society score (MSTS) [6] was recorded at final follow-up by an independent physiotherapist using the standard 30-point scale. Descriptive statistics only were used because of the small sample size, and STROBE (STrengthening the Reporting of OBservational studies in Epidemiology) guidelines were followed.

## Results

Patient demographics and baseline characteristics

Ten patients were included (mean age 29.7 years, range 20-38), of whom three were male, and seven were female. All presented with histologically confirmed Campanacci Grade III distal femoral GCT. The mean preoperative symptom duration was four months (range 2-8). Mean post-resection defect length was 13.5 cm (range 12-16 cm; SD 1.4). Right-sided involvement occurred in seven cases, left-sided in three cases. No patients had radiologically apparent pulmonary metastases at presentation.

Surgical outcomes

All patients underwent successful Ilizarov frame assembly and bone transport without intraoperative complications. Mean external fixation duration was 16.2 months (range 14-19, SD 1.8). Mean regenerate bone length was 10.9 cm (range 9.5-12.5 cm, SD 1.0). Docking-site union was achieved in all 10 patients at a mean of 6.2 months post-docking (range 5-8 months, SD 1.1). All frames were removed following clinical stability and radiographic consolidation (minimum three cortical bridges). External fixation index averaged 1.49 months/cm regenerate (range 1.36-1.52). Detailed patient data are shown in Table 1.

**Table 1:** Patient characteristics, complications, and outcomes (N=10) *Non-union: "cons" = conservative management; "Rail" = Rail Fixator revision + grafting (partial Ilizarov failure due to patient non-compliance) **Maximum score: 30 points MSTS: Musculoskeletal Tumor Society score; LLD: limb length discrepancy

Case	Age (year)	Sex	Defect (cm)	Side	Fixation (months)	Regenerate (cm)	Varus (>5°)	LLD (>3 cm)	Non-union*	Pin Infection	Fluid Collection	MSTS (/30)**
1	20	F	13	R	15	10	Yes	No	No	No	Yes	23
2	23	F	12	L	14	9.5	No	No	No	Yes	No	22
3	28	M	14	R	18	12	Yes	Yes	No	No	Yes	21
4	31	F	13.5	R	17	11	No	No	Yes (Rail)	No	No	26
5	38	F	16	L	19	12.5	Yes	Yes	No	Yes	Yes	24
6	33	F	13	R	14	10	No	No	No	No	No	25
7	27	M	12.5	L	16	10.5	No	No	Yes (cons)	No	No	23
8	25	M	13	R	15	10.5	No	No	No	No	No	23
9	35	F	14.5	R	17	11.5	No	No	No	Yes	No	25
10	29	F	15	R	19	11.5	Yes	No	No	No	No	24
Total	29.7±5.8	7F/3M	13.5±1.4	7R/3L	16.2±1.8	10.9±1.0	40%	20%	20%	30%	30%	23.1±1.4

Complications

Varus Angular Deformity

**Figure 2:**
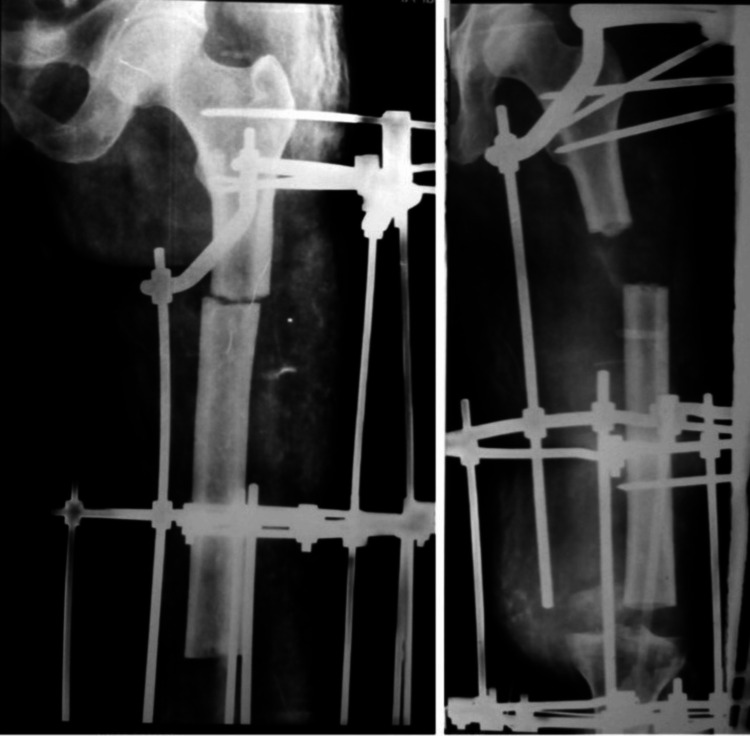
Progressive varus angulation of the transported segment

**Figure 3:**
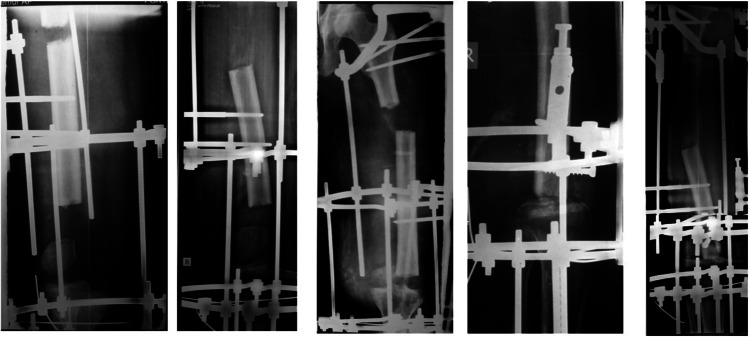
Serial X-rays of a patient during bone transport

**Figure 4:**
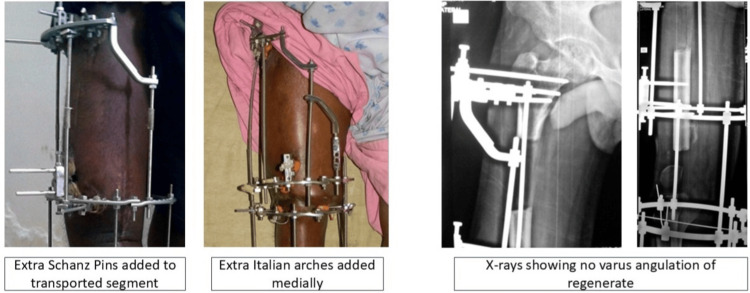
Corrective measures to prevent varus angulation

Varus angular deformity was detected in 4/10 patients (40%) during months 2-4 on monthly radiographs. Possible contributors included adductor magnus tension, suboptimal medial rod placement, and lateral soft tissue forces. (Figures 2, 3) Management comprised half-pin upsizing (5.5 to 6.0 mm), medial distraction (+2 mm), distal pin valgus orientation (10°), and periodic soft tissue releases. Three cases corrected fully; one retained mild residual varus (<10°, clinically non-progressive) (Figures 2-4 ). 

Limb Length Discrepancy

Limb length discrepancy >3 cm occurred in 2/10 patients (20%) and was managed with permanent shoe lifts (3-4 cm); both patients achieved comfortable adaptation without secondary lengthening.

Docking-Site Non-Union

**Figure 5:**
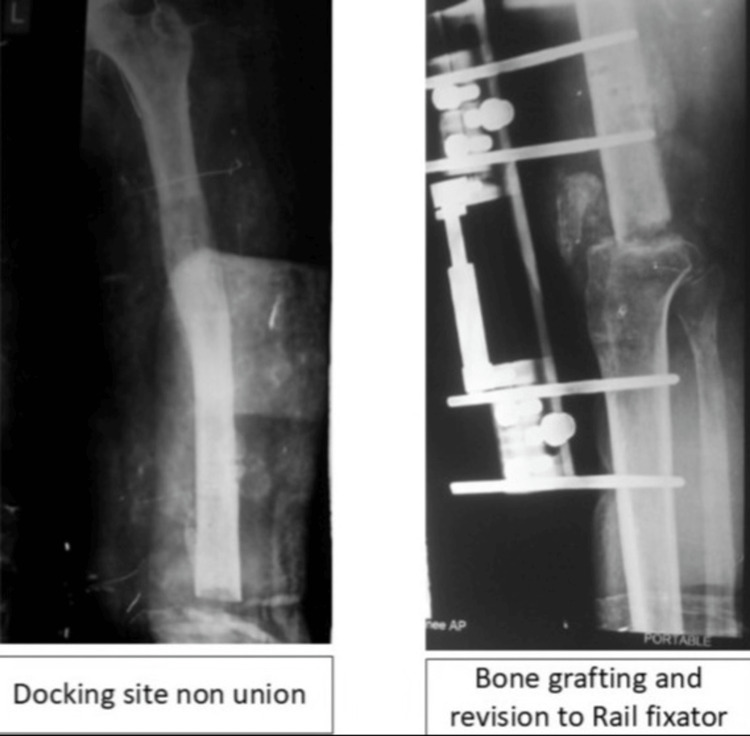
A case with docking site non-union managed with bone grafting and rail fixator application

Docking-site non-union is defined as >3 mm radiolucency at six months post docking and was found in 2/10 patients (20%). Case 7 resolved with prolonged compression (union at nine months total). Case 4 (10%) required iliac crest bone grafting plus monolateral Rail Fixator conversion due to patient/family non-compliance with circular frame after six months, achieving union at 10 months total (classified as partial Ilizarov failure requiring hybrid salvage) (Figure 5).

Pin-Tract Infections

Pin-tract infections occurred in 3/10 patients (30%), all limited to soft tissue involvement alone. Resolved with enhanced local care (chlorhexidine irrigation), temporary wire removal, and oral antibiotics (7-10 days). No deep infections or osteomyelitis were reported.

Docking-site Fluid Collection

Docking-site fluid collection occurred in 3/10 patients (30%) and was subacute seromas/hematomas (3-8 weeks post-docking, >10 mm on ultrasound). All resolved after a single aspiration plus compression bandaging.

Other Complications

Mild hardware loosening (n=2) was managed with frame realignment; superficial skin irritation (n=2) was resolved with topical care. No neurovascular injuries, hardware failure, or unplanned readmissions occurred.

Functional outcomes

At final follow-up (mean 34 months, range 25-48), all 10 patients achieved independent ambulation without walking aids. Mean MSTS score was 23.1/30 (77%, range 21-26, SD 1.4), assessed by an independent physiotherapist. Two patients (20%) required permanent shoe lifts for length discrepancy; mild gait abnormality persisted in these cases. No walking aids were required. No tumour recurrences, deep infections, or late complications occurred in the followed cohort during the observation period.

## Discussion

Reconstruction after tumour resection is guided by the size of the defect and the extent of soft tissue loss. Commonly used options include endoprosthetic replacement, osteoarticular allograft reconstruction, and arthrodesis, with the final choice depending on the patient’s activity demands, the surgeon’s judgment, and institutional resources, particularly in resource-limited settings.

An endoprosthesis restores knee stability and mobility immediately but carries a high cost and finite durability [7]. Aseptic loosening is a key failure mode [8]. Five‑year survival rates are about 73-83%, falling to 59-67% by 10 years [7,9]. More extensive resections with prosthetic reconstruction may increase infection risk, but supporting data are limited [10].

Vascularized fibular grafting can achieve good biological healing and graft hypertrophy, but it is technically demanding, and complications are not uncommon [11]. There are only a handful of reports on using the Ilizarov method for giant cell tumours near the knee [12,13]. Some authors suggest adding an intramedullary nail to bone transport procedures to reduce how long an external frame is needed [14]. In the present series, Ilizarov-mediated bone transport appears to be a useful limb-salvage option in selected young adults after distal femoral GCT resection, although the technique is associated with important mechanical and infectious complications.

Mechanical complications were prominent in this series, with varus deformity being the most frequent. Medial drift during femoral bone transport may be related to adductor forces and the biomechanical challenges of the femur. Early detection with monthly radiographs, timely frame adjustment, and counter-distraction were important in limiting functional impact. Mild residual axis deviation, when not associated with pain or instability, may be acceptable if union is achieved. Limb length discrepancy remained another concern, particularly when regeneration formation lagged behind the planned distraction. In our patients, shoe lifts were sufficient, and functional limitation was minimal. Factors such as patient intolerance for prolonged transport and premature docking may have contributed to incomplete length restoration.

Docking-site non-union remained a challenging complication, especially in sclerotic or biologically compromised bone. In this series, eventual union was achieved in all patients, but one case required conversion to a rail fixator with bone grafting. This should be regarded as a failure of the primary Ilizarov construct but a successful salvage strategy. Judicious autografting at the time of docking should be considered in high-risk defects, particularly when the metaphyseal ends appear sclerotic or when biological healing is uncertain. Pin-tract infection was another common issue, although all cases were superficial and responded to enhanced local care and oral antibiotics without progression to deep infection. This underscores the need for strict pin-site monitoring, patient education, and early intervention.

Early mobilization of adjacent joints, muscle strengthening, and gait training should begin as soon as tolerated and continued throughout the treatment period to maximize function and independence. Ongoing counselling and peer support can reduce anxiety, improve body image, and improve compliance with prolonged external fixation. The emotional burden of lengthy external fixation and altered limb appearance was reduced in our patients through structured psychological support. Overall, our findings suggest that acceptable functional outcomes can be achieved when patients are managed within a comprehensive rehabilitation program and are closely monitored for complications throughout treatment.

Comparison with alternative modalities

Endoprosthetic replacement offers immediate stability, but reported five-year survival is 73-83%, falling to 59-67% by 10 years, with aseptic loosening being the main mode of failure. Vascularized fibula grafts are more suitable for smaller defects of less than 10 cm but require microsurgical expertise. In contrast, Ilizarov reconstruction provides biologic durability without a permanent implant, though it usually requires a much longer treatment period, with average fixation lasting about 16 months compared with the four to six weeks of rehabilitation typical of prosthetic reconstruction. The complication pattern also differs, with Ilizarov methods more often associated with infection-related problems, whereas endoprosthetic reconstruction more commonly fails because of mechanical loosening.

Clinical implications and recommendations

Preoperatively, patients should be counselled in detail about the prolonged treatment course, the likelihood of complications, and the possibility of revision surgery, and the ideal candidates are motivated young adults with good soft tissue condition, no neurovascular compromise, and reliable family or social support. Technically, the use of stouter half-pins, medial rod reinforcement from the second month, and monthly radiographic surveillance beginning in month 2 may help reduce varus deformity and hardware-related problems; in selected cases with sclerotic docking ends, prophylactic iliac crest bone grafting should be considered, and treatment failure should be defined clearly as the need to convert to a non-circular fixation or persistent non-union beyond 12 months after docking.

Postoperatively, structured physiotherapy should begin early, pin-site care should be performed regularly with chlorhexidine-based cleaning, and psychological support should start from the first postoperative week to improve compliance and coping during the prolonged frame period. Follow-up should include monthly radiographs until frame removal, periodic clinical review after frame removal, and long-term oncologic surveillance with clinical examination every three months and chest radiographs every six months for up to five years to monitor for recurrence.

Study limitations

This study has important limitations. It is a retrospective case series with a small sample size, no control group, and no formal biomechanical or economic analysis. The reported mechanism for varus deformity remains a clinical hypothesis rather than a proven biomechanical fact. In addition, the findings may not be generalizable beyond young, selected patients treated in a resource-limited setting. Nevertheless, the series provides practical information on complication patterns and salvage strategies in a difficult reconstructive scenario.

## Conclusions

Ilizarov reconstruction enables limb salvage after wide distal femoral GCT excision in resource-constrained settings, achieving 100% union (with 10% hybrid salvage) and good function (MSTS 23/30). High but manageable complications demand vigilant multidisciplinary care. These preliminary descriptive findings support technique viability where alternatives are unavailable; larger prospective series with controls are needed to refine patient selection and optimize outcomes.
